# Development of Formaldehyde Biosensor for Determination of Formalin in Fish Samples; Malabar Red Snapper (*Lutjanus malabaricus*) and Longtail Tuna (*Thunnus tonggol*)

**DOI:** 10.3390/bios6030032

**Published:** 2016-06-30

**Authors:** Bohari Noor Aini, Shafiquzzaman Siddiquee, Kamaruzaman Ampon

**Affiliations:** Biotechnology Research Institute, Universiti Malaysia Sabah, Jln UMS, 88400 Kota Kinabalu, Sabah, Malaysia; noorainibohari@gmail.com (B.N.A.); amponk@ums.edu.my or profkamaru@gmail.com (K.A.)

**Keywords:** formaldehyde biosensor, glassy carbon electrode, gold nanoparticles, ionic liquid, methylene blue

## Abstract

Electrochemical biosensors are widely recognized in biosensing devices due to the fact that gives a direct, reliable, and reproducible measurement within a short period. During bio-interaction process and the generation of electrons, it produces electrochemical signals which can be measured using an electrochemical detector. A formaldehyde biosensor was successfully developed by depositing an ionic liquid (IL) (e.g., 1-ethyl-3-methylimidazolium trifluoromethanesulfonate ([EMIM][Otf])), gold nanoparticles (AuNPs), and chitosan (CHIT), onto a glassy carbon electrode (GCE). The developed formaldehyde biosensor was analyzed for sensitivity, reproducibility, storage stability, and detection limits. Methylene blue was used as a redox indicator for increasing the electron transfer in the electrochemical cell. The developed biosensor measured the NADH electron from the NAD^+^ reduction at a potential of 0.4 V. Under optimal conditions, the differential pulse voltammetry (DPV) method detected a wider linear range of formaldehyde concentrations from 0.01 to 10 ppm within 5 s, with a detection limit of 0.1 ppm. The proposed method was successfully detected with the presence of formalin in fish samples, *Lutjanus malabaricus* and *Thunnus Tonggol*. The proposed method is a simple, rapid, and highly accurate, compared to the existing technique.

## 1. Introduction

The fast urbanization of society has led to an accumulation of many toxic elements, especially carcinogens in the environment. Formaldehyde is considered as a toxic element since it has been classified as Group 1 carcinogen to human beings by the International Agency for Research on Cancer (IARC). Formaldehyde accumulates in the body and adversely affects lifespan. Formaldehyde is commonly used as bath treatment in aquaculture and the preservation of any biological samples. Nowadays, the fish vendors have learned to use formaldehyde, better known as formalin (40% formaldehyde), to preserve fish, as the chemical is a renowned food preservative. This has been proven by recent news and research, claiming the use of formaldehyde in fish preservation is very popular, particularly in Asian countries [1,2].

Fish and seafood are an important part of a healthy diet and are deliberated as the largest source of protein in Malaysia. By composition, fish contains fat, free amino acids, and water, which is susceptible to spoilage by microorganisms and biochemical reactions during *post mortem* processing [3]. The flesh of fish can spoil quickly and it should be eaten on the day of capture, unless cured [4]. Microbial and enzymatic activities in fresh fish can deteriorate the fish and alter the biological compounds under normal freezing storage. Thus, fish and seafood are very perishable and can only be kept fresh in ice for 8 to 14 days, depending on the species. In order to keep the freshness of fish and seafood, fishermen and fish vendors tend to carelessly use formaldehyde as a preservation agent because formaldehyde is well-known in food preserving. However, the dose of formaldehyde used in the fish preservation activity has yet to be disclosed. Lack of knowledge on the lethal effect of formaldehyde has led to its misuse.

The formaldehyde mechanism of action for fixing lies in its ability to form cross-links between soluble and structural proteins. The resulting structure retains its cellular constituents in their in vivo relationships to each other, giving it a degree of mechanical strength which enables it to withstand subsequent processing, as reported by Environmental and Occupational Health and Safety Services, 2004 [5].

The limitation on exposure and health risk towards this hazardous substance in occupational settings has been set by the Occupational Safety and Health Administration and Environmental Protection Agency. According to the United States Environmental Protection Agency (EPA), maximum daily dose reference (RfD) for formaldehyde is 0.2 µg·g^−1^ body weight per day [6]. According to Malaysian Food Regulations 1985, Regulations 148 and 159 (2006), only smoked fish and meat are permitted to incidentally absorb formaldehyde during processing in a proportion not exceeding 5 μg·g^−1^ [7]. However, for fresh fish, the permitted amount of formaldehyde is not yet specified [8].

Standards have been fixed by the Occupational Safety and Health Administration, Malaysian Food Regulations, as well as by the EPA, to limit human exposure and health risk in occupations involving formaldehyde use. Formaldehyde levels must be accurately monitored to act in accordance with these standards. Many conventional methods are available for determination of formaldehyde concentration levels in the laboratory, such as gas chromatography-mass spectrometry (GC-MS), high-performance liquid chromatography (HPLC), fluorimetry, Nash test, gravimetric methods, and other chemical-based biosensors [8,9,10,11]. Colorimetric detection methods, such as Deniges’ method, and Eegriwe’s method, have been known since the beginning of the 20th century [6]. Unluckily, these methods, reagents, and reaction products are often just as harmful to human health. All of these conventional methods require similarly hazardous reagents and suffer from a number of interferences, resulting in false positives. Additionally, these methods are impracticable for real-time measurements because of the required time for apparatus setup. Recent efforts have turned toward the development of biological methods of detection combined with physical transducers, and biosensors. Electrochemical-based biosensors enable direct, reliable, and reproducible measurements. The developed formaldehyde biosensor is a simple sample preparation procedure, resulting in faster response, lower cost, zero pollution, and user friendliness.

Determination of formaldehyde in Indian mackerel (*Restrelliger kanagurta*) was previously detected with different levels of formaldehyde using electrochemical biosensors which were reported by Marzuki et al. [2], it lacks performance regarding the nanomaterials-based detection of formaldehyde in fish samples. Owing to the gap, this study is a stepping stone for the research on formaldehyde determination in fresh fish samples using nanomaterials.

**Scheme 1:**
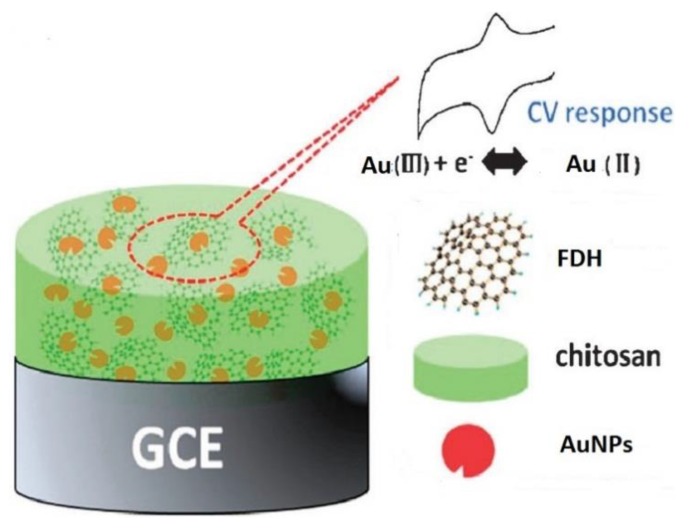
The interaction of formaldehyde dehydrogenase, chitosan, and Au nanoparticles on a glassy carbon electrode.

**Scheme 2:**
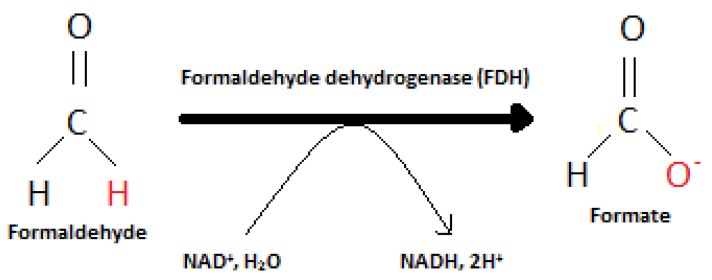
Scheme of a formaldehyde biosensor. Formaldehyde is oxidized into formate and produces NADH as byproduct. The amount of NADH can be measured by the electrode.

For this study, the electrochemical biosensor was developed based on the gold nanoparticles (AuNPs), ionic liquid [EMIM][Otf], chitosan (nanocomposite membrane), and glassy carbon electrode for determination of formaldehyde (Scheme 1 and Scheme 2). Formaldehyde dehydrogenase used as bio-recognition receptor in the system, so any interference can be avoided (selective towards the substrate, formaldehyde). Immobilization of the enzyme is applied using chitosan, which acts as a binder. Immobilization could enhance the electro-catalytic properties of the electrode or, in other words, increase the rate of chemical reactions without being consumed in the process (decrease the fouling effects) and obtain better results.

A formaldehyde biosensor (FDH) was initially immobilized onto the surface of chitosan, which was employed to hydrolyze formaldehyde with the production of formic acid and NADH, as displayed in Equation (1). FDH immobilized onto chitosan has been investigated, with respect to the effects of response time, pH range, scan rate, and formaldehyde concentration on the hydrolysis of formaldehyde.

(1) $$\text{HCHO}+{\text{NAD}}^{+}+H_{2}O\underset{Formandehyde\;dehydrogenase}{\to }\text{HCOOH }+\text{ NADH }+{\text{ H}}^{+}$$

The use of chitosan (CHIT) film for enzyme immobilization has low cost, zero toxicity [12,13], and is easily controlled by pH manipulation [14]. Nanoparticles (AuNPs) are commonly immobilized onto chitosan along with formaldehyde (FDH) enzymes to provide large surface areas for a higher electrochemical reaction rate [15]. This research has included ionic liquids in the electrochemical electrode to enhance the high sensitivity and homogenous deposition. FDH is found to be specifically reactive to formaldehyde [16], whereas the presence of a cofactor NAD^+^ will provide higher stability of the reaction.

The aim of the project was to develop an electrochemical biosensor using formaldehyde dehydrogenase (FDH) and chemically-modified electrode based on nanomaterials-coated with chitosan. Since there are a very limited number of techniques for the real-time determination of formaldehyde on the real fish sample, it is important to investigate the formaldehyde content in the fish in real-time since it is claimed to be the major contaminant in fish, in order to understand better the risks of fish consumption, to manage the risks of consumption and to provide additional information in food safety.

## 2. Materials and Methods

### 2.1. Chemical and Materials

Formaldehyde dehydrogenase (EC 1.2.1.46) collected from *Pseudomonas putida* (specific activity 1.6 U/mg) and β-nicotinamide adenine nucleotide (NAD^+^) obtained from Sigma (Spruce Street, St. Louis, USA). Methylene blue (MB) was purchased from Sigma (Spruce Street, St. Louis, USA). Formaldehyde (37%) and acetic acid were purchased from Merck company. Gold nanoparticles (AuNPs) and ionic liquid [EMIM][OTF] were purchased from Sigma (Spruce Street, St. Louis, USA). All chemicals used in the experiments were analytical reagent grade. Deionized water was obtained from a Millipore Milli-Q purification system. The Malabar Red Snapper *(Lutjanus malabaricus)* and Longtail Tuna *(Thunnus Tonggol)* samples were collected from the three different local wet markets in Kota Kinabalu, Sabah, Malaysia.

### 2.2. Apparatus and Equipments

An electrochemical study was carried out using a Metrohm AutoLab Eco-chemie, Netherlands, with the software package NOVA 1.8. A Metrohm 3 mm glassy carbon electrode (GCE) was used as the working electrode to be coated with the enzyme and nanomaterials onto the membrane via covalent immobilization. An Ag|AgCl|KCl 3M electrode, referred as the reference electrode, and a platinum (Pt) wire were employed as a counter-electrode.

### 2.3. Preparation of Reagents

Stock solution of MB (1 mM) was prepared in a 50 mM Tris–HCl (pH 7.0) solution. The diluted solutions were prepared by an appropriate dilution with the Tris–HCl buffer. The FDH mixed thoroughly with chitosan, gold nanoparticles (AuNPs), and ionic liquid ([EMIM][OTF]), the mixtures were homogenized for 24 h with 3000 rpm. The pH (50 mM Tris-HCl buffer) was determined using a pH meter, model PC 2700-meter kit. Before use, the pH meter was calibrated with standard buffer solutions of pH 4, 7, and 10. The amounts of chemicals were weighed accurately by using a digital electronic balance, model GR-200. For the preparation of NAD^+^ solution (0.5 mM) 2.5 µL of NAD^+^ solution (5 mM) was dissolved in 25 mL of deionized water. Standard formaldehyde stock solution (100 ppm) was prepared by adding 25 mL deionized water to 6.8 µL of formaldehyde (37%) solution. Different concentrations of standard formaldehyde were prepared on the range of 0.01–10 ppm. All of the solutions were prepared fresh at the beginning of each experiment.

### 2.4. Preparation of the FDH/AuNPS/([EMIM][OTF])/CHIT Modified Electrode

Before modification, pre-treatment of GCE was performed according to Siddiquee et al. [17] procedure with some modifications. The glassy carbon electrode (GCE) was polished with 0.05 µm alumina slurry for 2 min on a smooth cloth in order to make sure the surface is cleaned of any foreign residues. Then, the electrode was ultrasonicated in an ultrasonic bath for 15 min and dried at room temperature. For immobilization of FDH with chitosan, FDH solution (30 mg/mL) was prepared by dissolving 6 µL of stock FDH solution (50 mg/mL) in 10 µL of 0.05 mM buffer (pH 7.0), which corresponded to the optimum pH. The FDH solution was thoroughly mixed with the chitosan in a volume ratio of 1:20 and sonicated for 15 min. Then, 10 µL of the mixture was deposited on the surface of the GCE and allowed to evaporate at room temperature. This procedure resulted in the fabrication of homogenously-dispersed FDH/CHIT immobilized on the glassy carbon electrode surface.

An appropriate amount of the AuNPs was dispersed on the CHIT. Then it was sonicated for 20 min after stirring for 8 h. The mass ratio of AuNPs: CHITwas 1:5. A 20 µL of FDH solution in a Tris-HCl (pH 7.0, 0.05 M) was added. An ionic liquid was dispersed in the AuNPs/CHIT composite, and then sonicated for 3 h to produce homogeneous suspension. The ratio of ionic liquid was fixed at 3% (*v*/*v*) in the experiments. Methylene blue (MB) was accumulated onto the membrane surface by immersing the electrode and stirred into 10 mL of MB for 2 min without applying any potential. After accumulation of MB, the electrode was rinsed with buffer (pH 7.2, 0.5 M) for 2 min to remove any non-specifically-bound MB. It was then transferred into an analytical buffer added with substrate in an electrochemical cell for cyclic voltammetry and differential pulse voltammetry measurements.

### 2.5. Assembly of Electrochemical Biosensor

FDH-immobilized chitosan was deposited on the surface of a Thermo-Orion glassy carbon electrode and kept in a steady position by an O-ring. The electrode was dried at room temperature for at least 4 h. The counter and reference electrodes were immersed together into a stirred reaction media containing phosphate buffer solution. A 5 mL of substrate was injected into the reaction media and the accumulation products signal was measured and processed by the AutoLab Nova 1.8 software package. The same protocol was applied to the unmodified GCE.

### 2.6. Reproducibility Assay

Reproducibility was associated with precision, where the functional ability of the electrode was kept in an acceptable range, especially when the electrodes were produced in large quantity [1]. Individually, five different working electrodes were prepared and analyzed the reproducibility by using the same concentration of formaldehyde solution, five times. 

### 2.7. Detection of Formaldehyde in Fish Samples

Fresh Malabar red snapper fish samples were collected from wet market in Kota Kinabalu, Sabah. The samples were stored at 4 ± 1 °C. Formaldehyde extraction procedure exactly followed the previously established method reported by Marzuki et al. [1]. The fish was thawed and only the flesh was taken and cut into small pieces. A 15 g of the flesh was homogenized with 30 mL of Tris–HCl (pH 7, 0.5 M) for 5 min, and the aliquots (a portion of a total amount of solution) were filtered. The filtration was done using Whatmann 1 filter paper. The sample was directly used without further extraction and treatment. Formaldehyde was determined using the modified electrode and the storage stability was measured in a real sample from 0 to 35 days at 4 °C. The experimental works were conducted with five replicates. Recovery assays were analyzed with varying storage periods.

## 3. Results and Discussion

### 3.1. Morphological Characterization of Modified GCE

#### 3.1.1. Morphological Analysis via Scanning Electron Microscope

**Figure 1:**
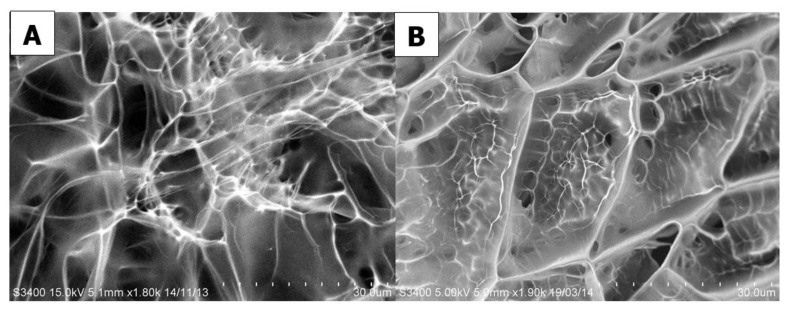
A scanning electron micrograph of (**A**) chitosan, CHIT; and (**B**) chitosan immobilized with Au nanoparticles and formaldehyde dehydrogenase, FDH/AuNPs/[EMIM][Otf]/CHIT/GCE.

The surface morphological analysis of the CHIT, AuNPs/CHIT, and AuNPs/[EMIM][OTF])/CHIT were observed by scanning electron microscope (SEM). Figure 1A shows the microporous protein fiber of the chitosan membrane. Based on the results, AuNPs were well dispersed, spherical in shape on the chitosan, and appeared as white dots on the protein fiber of the chitosan. FDH attached well with the AuNPs/[EMIM][OTF] net fiber matrix as shown in Figure 1B. Additionally, the image clearly illustrated that FDH molecules were scattered on the whole surface of AuNPs/[EMIM][OTF]/CHIT. When FDH was immobilized on the membrane fibers, the rougher fibers appeared with clusters of lumps of FDH as compared to without, using FDH on chitosan. The fibers of FDH/AuNPs/[EMIM][OTF]/CHIT were found rougher than that of the FDH/CHIT. The protein fibers were covered with many small spherical nanoparticles which increased the surface area of the chitosan. In the presence of [EMIM][OTF], it appears as a smooth surface and recovers the conductivity of modified GCE [18]. Additionally, [EMIM][OTF] acts as an efficient solvent in the electrochemical sensor. [EMIM][OTF] is an important source of ion transporters to transport charges in the nanomaterials. The conductivity performance of the biosensor containing AuNPs/[EMIM][OTF]/CHIT remarkably enhanced the potential current due to the presence of nanoparticles and ionic liquid contributions. From these results, it is strongly concluded that the combination of FDH and AuNPs are successfully immobilized on the chitosan for the development of a formaldehyde biosensor. The interaction of chitosan with AuNPs leads to the formation of cross-linked chitosan and nanoparticles [19]. Thus, the FDH/AuNPs/[EMIM][OTF]/CHIT structure has provided a large effective surface area for excellent adsorption and substrate diffusion, which is a critical issue for improving the response properties of the electrochemical enzyme sensor.

#### 3.1.2. Energy Dispersive Spectroscopy (EDS) Spectrum

**Figure 2:**
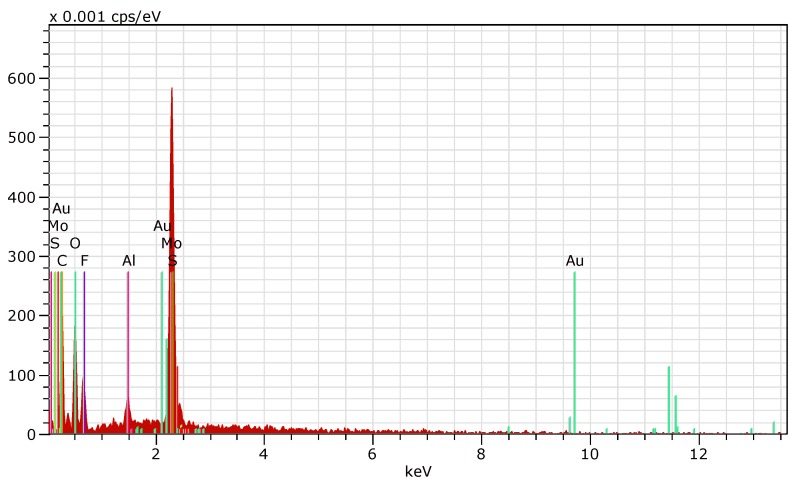
EDX spectrum of FDH/AuNPs/[EMIM][Otf]/CHIT.

The EDX spectrum in Figure 2 is a plot of X-ray counts vs. energy (keV). Energy peaks correspond to various elements in the modified membrane since it determines the abundance of specific elements. They are narrow and readily resolved, but many elements yield multiple peaks. The analysis indicates that the membrane has the phase structure and composition of the rutile form of FDH/NPs/([EMIM][OTF])/CHIT. Carbon (C), oxygen (O), and gold elements (Au) showed strong peaks. Elements in low abundance will generate X-rays that may not be resolvable from the background radiation. There are energy peak overlaps among different elements, particularly those corresponding to X-rays generated by emission from different energy level shells (C and O) in different elements. In the spectrum, there were closed overlaps of C or O and various lines with Au. Particularly at higher energy, individual peaks may correspond to several different elements. Therefore, there is an EDX spectrum peak of Au at 280 keV. Since lower atomic number elements have fewer filled shells, they have fewer X-ray peaks. Carbon, for example, only has one peak at 40 keV. Conversely, the higher atomic numbered elements have a greater number of X-ray peaks. While some of the high atomic numbered X-rays can be over 50 keV, a spectral range of 0–20 keV can detect all of the elements, as summarized in Table 1. From these results, it is strongly concluded that the combination of FDH and AuNPs are successfully immobilized on the chitosan for fabrication of the formaldehyde biosensor. The interaction of chitosan with AuNPs leads to the formation of cross-linked chitosan and nanoparticles as mentioned before.

### 3.2. Optimization Condition for Formaldehyde Determination

**Figure 3:**
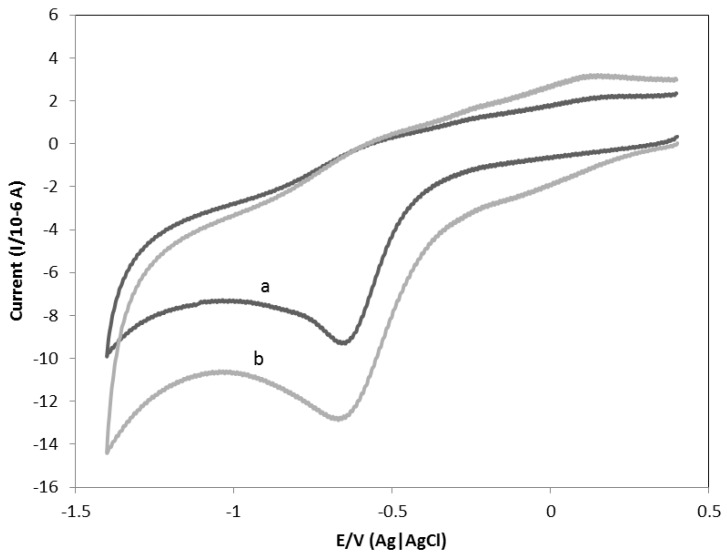
A cyclic voltammogram of a bare electrode (**a**) without being soaked in methylene blue; and (**b**) soaked in methylene blue (0.1 M of MB, in 10 ppm of formaldehyde).

To study the effects of using a redox reaction indicator, the unmodified glassy carbon electrode was soaked in a methylene blue solution for 5 min before running the analysis with known concentrations of the formaldehyde solution. The comparison was done after running the analysis with the presence and absence of the methylene blue solution (Figure 3). The methylene blue mediator is strongly retained inside the membrane through hydrophobic interaction. The first step was conducted using an unmodified electrode for examination with the effects of using a redox indicator. Then, the unmodified electrode was applied for an examination of the scan rate, pH, and interaction time, as well as the response range of formaldehyde.

The cathodic peak of bare GCE accumulated with 1 mM methylene blue (MB) increased, as compared to the cathodic peak currents, in the range of −9.30 × 10^−7^ to −12.89 × 10^−7^ A. 8 ppm of formaldehyde concentration was significant in the presence of MB as a remarkable marker for current signal. Determination of formaldehyde depends on the electrode modification components and the presence of mediator. Direct electron transfers between enzyme and electrode require a mediator since the transformation of electrons occur at a leisurely rate. MB is a cationic dye which has a reserved potential between 0.08 and −0.25 V in pH 2–8 solution. Hence, it is close to the potential used in this project. MB diffuses into CHIT fiber by electrostatic interaction [20]. According to Yao et al. [19], the role of the mediator is to shuttle electrons effectively between the electrolyte in the electrochemical cell and the bioactive center of the electrode.

The optimum working conditions were studied at 0.10 Vs^−1^ (voltage at which maximum current was generated) in analytical buffer, at pH 7.0. Different concentrations of formaldehyde were detected in the range of 0.01 to 10.0 ppm. The current signals of the enzyme reaction were measured using cyclic voltammetry (CV) and differential pulse voltammetry (DPV). All measurements were completed with three replications.

#### 3.2.1. Scan Rate Effects

**Figure 4:**
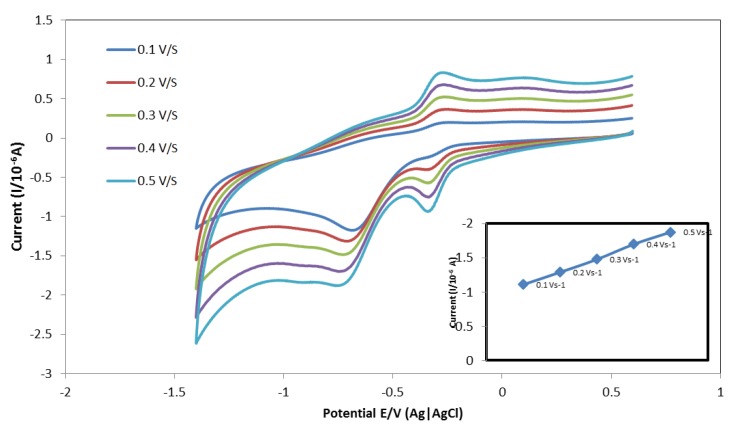
The cyclic voltammogram of the scan rate effects on the detection of formaldehyde. Scan rates used were 0.1, 0.2, 0.3, and 0.5 V/s. All were performed at room temperature, 25 ± 1 °C, and with the same concentration of formaldehyde, 8.0 ppm.

Useful information involving electrochemical reactions can be obtained from the relationship between the cathodic peak current and the scan rate. The kinetics of the bioactive center GCE reaction was investigated by studying the effects of the scan rate on the peak currents. The influence of scan rate on reduction of NAD^+^ at the GCE surface was investigated in the range of 0.10–0.50 Vs^−1^ by cyclic voltammetry (CV) (Figure 4). The peak current increased with an increasing scan rate. As can be seen, increasing the scan rate let anodic and cathodic peak potentials shift towards the positive and negative directions, indicating a charge transfer kinetics limitation. The higher scan rate gave fragmented peaks and the potential range was shifted to the more negative region, which means it requires a larger number of voltages per second. Both the anodic and cathodic peak currents were increased linearly, proportional to the scan rate ranging from 0.04 to 0.10 Vs^−1^. The overall redox process is focused at the electrode surface, which can be considered to be relatively fast on the voltammetry time scale, indicating a surface-confined redox process and corresponds to the rapid conversion of a surface electrode without a diffusion-controlled reaction step. Therefore, 0.1 Vs^−1^ was selected as the optimum scan rate. The reaction was stable at a low scan rate; therefore, 0.1 Vs^−1^ was chosen and applied, similarly to Marzuki et al. [4].

#### 3.2.2. pH of Analytical Electrolyte Effects

**Figure 5:**
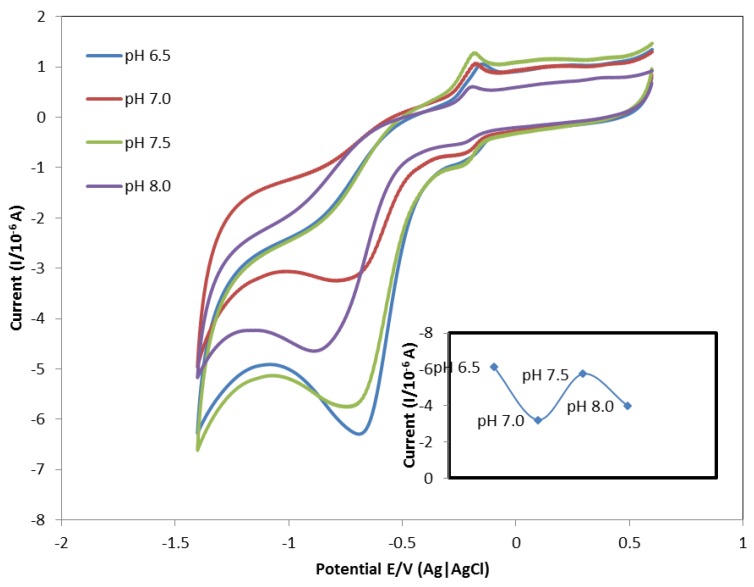
The cyclic voltammetry of effects of pH of the analytical buffer. The pH of the buffer used were 6.0, 6.5, 7.0, 7.5, and 8.0. All were performed at room temperature, 25 ± 1 °C, and with the same concentration of formaldehyde, 8.0 ppm.

The pH value of the electrolyte is important for the performance of the electrochemical biosensor. Therefore, the effects of the pH of the supporting electrolyte were studied from 6.0 to 8.5 at intervals of 0.5 using the Tris–HCl buffer (50 mM). The response current increased and reached a maximum at pH 6.5 and decreased obviously, as shown in Figure 5. The cathodic peak potentials seem to be intensely dependent on the pH of the electrolyte. A linear negative shift was observed in the cathodic peak current by increasing the pH value. The results showed that the oxidation of formaldehyde involves a dissimilar number of transferred H^+^ or OH^−^ at various pH values, which led to a different response, similarly reported previously by Lei et al. [21]. The peaks drop at pH 7.0–8.0 due to deprotonation of ions occurred. The obtained results were similar to those previously reported by Qian et al. [22]. The acidic environment enhances the reaction because H^+^ is needed for NAD^+^ to reduce to NADH. In fact, the immobilized FDH can retain its activity under broad pH conditions, indicating that the membrane and AuNPs are provided a favorable biocompatible microenvironment for the survival of FDH. Thus, pH 6.5 was selected as the optimum pH to obtain maximum sensitivity and bioactivity and used throughout the studies.

#### 3.2.3. Interaction Time Effects

**Figure 6:**
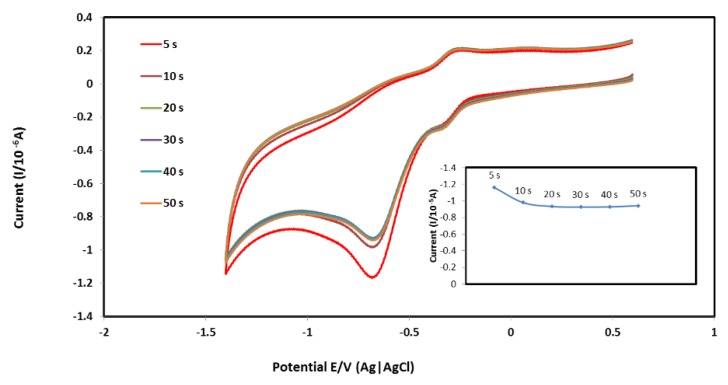
The cyclic voltammetry of effects of interaction time for detection of formaldehyde. The interaction times of the biosensor used were 5, 10, 20, 30, 40, 50, and 60 s. All were performed at room temperature, 25 ± 1 °C, and with the same concentration of formaldehyde, 8.0 ppm.

The interaction time of formaldehyde dehydrogenase and the substrate were influenced the the performance of the GCE in the presence of MB. The electrochemical cell was obtained from about 8 ppm of standard formaldehyde with 0.05 mM Tris-HCl buffer (pH 7.0). The interaction time was from 5 to 50 s and the scan rate fixed at 0.10 Vs^−1^ (Figure 6). The maximum current response was found at 5 s, implying that the accumulation of FDH and the substrate were saturated at the surface of the bioactive center of GCE. At 5 s, a complete enzymatic reaction was reached. There was sufficient time within 5 s to transform the formaldehyde to formic acid and reduced NAD^+^ to NADH. The cathodic peak value increased slowly and flattened with time; thus, 5 s was chosen as the optimum interaction time for the determination of formaldehyde.

### 3.3. Formaldehyde Detection

**Figure 7:**
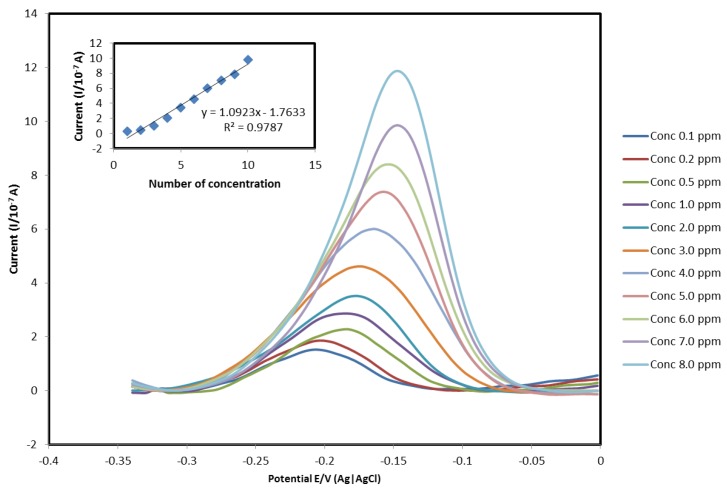
The diffrential pulse voltammogram of different concentrations based on FDH/AuNPs/[EMIM][Otf]/CHIT/GCE, conducted at pH 6.5 and temperature of 25 ± 1 °C. The concentrations of formaldehyde used were in between range 0.1–10 ppm.

The electrochemical behavior of the bare GCE and modified GCE were characterized by DPV using MB as an electroactive indicator (Figure 3). DPV was employed for the quantitative determination of formaldehyde, which is relatively sensitive compared to CV analysis. The shifting of the peak depended on many factors, such as scan rate, interaction time between enzyme and substrate, pH, and concentration of substrate. The developed formaldehyde biosensor was conducted using the modified FDH/AuNPs/[EMIM][Otf]/CHIT to react with different concentrations of formaldehyde as shown in Figure 7. Under optimum conditions, the DPV method was detected with different concentrations of formaldehyde in the range from 0.01 to 10 ppm, with detection limit of 0.1 ppm (*n* = 5). Due to the presence of AuNPs and ionic liquid ([EMIM][Otf]), large surface areas of the electrode for immobilizing homogenous formaldehyde were established. The performances of the constructed formaldehyde biosensors are compared and the results are shown in Table 2. The results indicate a good analytical performance for the development of a formaldehyde biosensor.

### 3.4. Mechanism of the Formaldehyde Biosensor

In this reaction, formaldehyde dehydrogenase acts as the electron transfer to facilitate the addition of one hydrogen atom to NAD^+^ and reduced it to NADH, whereas formaldehyde converted to formic acid. In fact, cofactor NAD^+^ avoided the blocking of O_2_ from electrocatalytic oxidation. The compact combination of FDH/AuNPs/([EMIM][OTF])/CHIT with the electrode surface enhanced the transfer speed of electrons and further increased the catalytic activity of formaldehyde due to AuNPs properties which have high biological compatibility, high catalytic efficiency, strong adsorption ability, a fast electron transfer rate, and easy preparation. The cathodic current of formaldehyde (started at about −0.5 V vs. Ag|AgCl) decreased with increasing formaldehyde concentration until 10 ppm, indicating that the consumption of oxygen over the course of the enzymatic reaction of FDH with formaldehyde. The majority of the immobilized FDH molecules are responsible for the formaldehyde conversion and reduction of NAD^+^.

Nanoparticles commonly act as a semiconductor material which are used as a supportive material in the development of an electrochemical biosensor. The modified AuNPs/[EMIM][OTF]/CHIT electrode displayed good biocompatibility and excellent electrochemical conductivity. As a result, the use of composite materials based on the integration of the membrane with some other materials to combine properties of the individual components has gained increasing attention [27]. Consequently, the immobilization of the FDH provided the highest signal response, which indicated that rapid increases of the peak current of MB accumulated due to the interaction of FDH and the substrate on the electrochemical cell. Due to some special characterization of ionic liquids, such as wide potential gaps (a voltage range between which the electrolyte is not oxidized or reduced) and high electrical conductivity, hydrophobicity, and insolubility in water. The reduced FDH donates the excess electron to the AuNPs/[EMIM][Otf]/CHIT together with the MB nanocomposite membrane to reduce the redox property. The MB oxidizes by transferring the electron to the external circuit due to efficient electron transfer and the good redox property of the prepared nanocomposite bio-membrane.

The direct reduction of NAD^+^ at the bare electrode is not suited for analytical application due to the slow electrode kinetics and low potential material, so preparation of a modified electrode with catalytic functionality is of practical significance. Since the electro-catalytic property of the modified electrode is obviously affected by the physical and chemical characteristics of the modifiers on the electrode surface, designing a membrane preparation method is essential. The factors are evaluated by electrochemical techniques based on the affected process of modification and chitosan property. The main advantage of using NAD^+^ dependent dehydrogenase-based biosensors is that oxygen (O_2_) does not interfere in formaldehyde detection because it does not participate in the reaction.

Rinaudo [14] has reported that chitosan is the only pseudo-natural cationic polymer in which the NH_2_ can be added with positive charge to increase solubility, while the polysaccharide has several ionizable groups that can act as a polyelectrolyte in acidic media. This unique characteristic has made the condition of chitosan be easily manipulated through pH control. FDH could be incorporated into AuNPs/CHIT via electrostatic forces. Au nanoparticles are characterized by a porous structure with hundreds of empty channels. It has reported that proteins are absorbed onto the membrane through binding of FDH’s carbonyl groups interacting with AuNP’s vicinal hydroxyl groups via electrostatic interaction [28]. The electrostatic interaction between oppositely-charged membranes (CHIT) and nanomaterials (ionic liquid and AuNPs) are usually strong and stable. The compatibility of AuNPs makes it excellent material for enzyme immobilization. Additionally, immobilized enzymes are more stable and easier to be reused than free enzymes.

In summary, CHIT has been used for the effective immobilization of molecules through electrostatic attraction in order to improve the stability and deposited onto the GCE. AuNPs increased the surface area of immobilization. Meanwhile, the ionic liquid [EMIM][Otf] acts as an ionic solvent which diffuses easily due to its wide solubility in the solutions, especially during electrochemical measurements. Combining all of the characteristic above, FDH/AuNPs/[EMIM][OTF]/CHIT indicated great potential in electrochemical activity for formaldehyde detection. Thus, the developed electrochemical method was detected with different concentration via the change in potential values.

### 3.5. Reproducibility Assay

**Figure 8:**
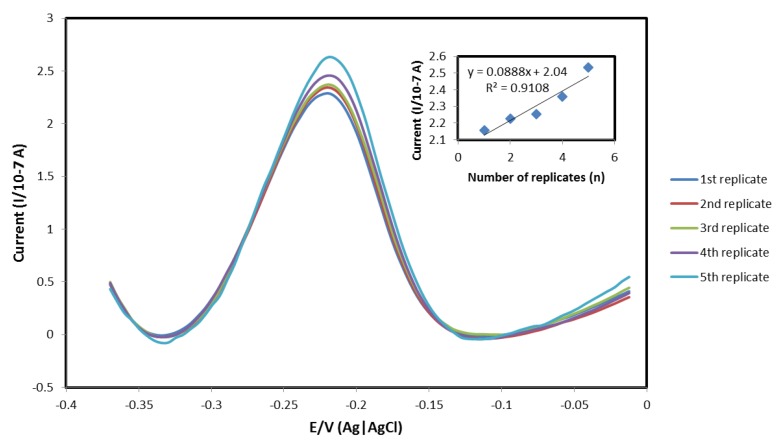
The reproducibility patterns of constant concentration of formaldehyde based on FDH/AuNPs/[EMIM][Otf]/CHIT/GCE was performed at pH 6.5 and temperature of 25 ± 1 °C. The concentration of formaldehyde used was 8.0 ppm.

A reproducibility assay is ian mportant characterization of the biosensors, showing whether the biosensor can be reproduced using the same materials and procedures. The reproducibility of the modified FDH/AuNPs/[EMIM][Otf]/CHIT/GCE in formaldehyde detection are almost similar (*p* < 0.05), implying that the reproducibility of such electrodes are high. A similar reproducibility assay was observed with different concentrations (1, 4, and 8 ppm) of formaldehyde (Figure 8). The response of the biosensors developed is reproducible, and the relative standard deviation of sensor responses below 1% (*n* = 10). Based on the result, the hypothesis is accepted where the reproducibility of the modified electrodes is high.

### 3.6. Real Sample Analysis

#### Spike Recovery Test and Stability

In order to validate and verify the applicability of the developed biosensor, the detection of formaldehyde in some fish samples were studied. The electrochemical method was applied to two different fish samples, which are Malabar Red Snapper (*Lutjanus malabaricus)* and Longtail Tuna *(Thunnus tonggol*). The recovery rate was calculated to be 81.2% to 82.2%, showing the high accuracy rate of the developed method. It showed good standard relative deviation (RSD) which is less than 1% (Table 3). The spike recovery test was performed to validate the accuracy of sample values from invalidated fish samples. A blank *Lutjanus malabaricus* and *Thunnus tonggol* were spiked with 8.00 ppm of formaldehyde. According to Mathias et al. [23], the recovery percentage should be above 80% to represent high accuracy. The stability is one of the most important characteristics for commercial application of any biosensor system. The operational stability test demonstrated that the steady state response did not decrease for at least 7 h (corresponding to approximately 70 measurements). Furthermore, the developed biosensors showed good recovery rate stability in 50 mM of phosphate buffer, at pH 6.5; the response remaining stable for more than one month for all types of bio-recognition elements used.

## 4. Conclusions

Electrochemical methods have proved to be an effective and inexpensive way for monitoring formaldehyde content due to their high sensitivity, reliability, rapidity, and economy. An novel electrochemical biosensor is developed based on FDH/AuNPs/[EMIM][Otf]/CHIT/GCE using MB as a redox indicator. AuNPs are dispersed onto chitosan and ionic liquid, which have enhanced the surface for the immobilization of FDH which increases the detection sensitivity of formaldehyde. The results indicated that the AuNPs provided a nano-sized environment for the close interaction between the enzyme (FDH) and the modified electrode, which are essential for efficient direct electron transfer. The developed formaldehyde biosensor has several advantages over conventional method such as good sensitivity and stability. The detection limit is 0.1 ppm, with linear coefficiency (*r^2^*) of 0.9787 and recovery rate from 81.2% to 82.2%. These results suggested that the developed biosensor offers a simple, rapid, sensitive, highly selective, wide detection range, and a convenient method for formaldehyde monitoring in the fish-based research industry.

## Figures and Tables

**Table 1:** Chemical analysis of the energy-dispersive X-Ray (EDX) spectrum.

Element	Series Unn (wt.%)	C Norm. (wt.%)	C Norm. (at.%)	C Error (1 Sigma) (wt.%)
Carbon	K-series 24.90	26.64	45.49	4.92
Nitrogen	K-series 6.28	6.72	9.84	2.26
Oxygen	K-series 21.52	23.03	29.52	4.21
Fluorine	K-series 6.88	7.37	7.95	1.76
Aluminium	K-series 0.53	0.57	0.43	0.08
Sulfur	K-series 5.34	5.71	3.65	0.26
Gold	L-series 28.02	29.98	3.12	4.26
	Total	93.46	100.00	100.00

**Table 2:** Comparison of analytical performance of various formaldehyde biosensors.

References	Range of Concentration	Detection Limit	Mode of Operation	Enzyme	Mediator
[20]	1.3 ppb–1.2 ppm	-	Amperometry (E_ap_ = 800 mV)	FDH	-
[23]	0.3 ppb–1.5 ppm	0.3 ppb	Amperometry (E_ap_ = 800 mV)	FDH	Os polymer
[23]	-	-	Amperometry (E_ap_ = 800 mV)	FDH	Tetrathiafulvalene
[24]	1.5–90 ppm	22.8 ppm	Amperometry (E_ap_ = 800 mV)	AOX + FDH	-
[24]	30–210.2 ppm	22.22 ppm	Amperometry (E_ap_ = 800 mV)	AOX + FDH	DCIP
[25]	9.09–120.12 ppm	8.1 ppm	Amperometry (E_ap_ = 800 mV)	AOX	-
[25]	0.1–100 µM	0.1 µM	Amperometry (E_ap_ = 300 mV, pH 7.5)	FDH	-
[26]	1.8–15.4 ppm	9 ppm	Potentiometry	AOX	-
[26]	1.8–15.4 ppm	1.8 ppm	Amperometry (E_ap_ = 100 mV, pH 7.5)	AOX	-
[4]	1–10 ppm	1 ppm	Electrochemical	FDH	-
This study	0.01–10 ppm	0.1 ppm	Electrochemical (E_ap_ = 100 mV, pH 6.5)	FDH	Methylene blue

**Table 3:** Properties of recovery rate.

Samples	Recovery (%)	RSD (%)
*Lutjanus malabaricus*	81.2	0.64
*Thunnus tonggol*	82.2	0.32
